# Prenatal exposure to bisphenol A and autistic- and ADHD-related symptoms in children aged 2 and5 years from the Odense Child Cohort

**DOI:** 10.1186/s12940-021-00709-y

**Published:** 2021-03-12

**Authors:** Julie Bang Hansen, Niels Bilenberg, Clara Amalie Gade Timmermann, Richard Christian Jensen, Hanne Frederiksen, Anna-Maria Andersson, Henriette Boye Kyhl, Tina Kold Jensen

**Affiliations:** 1 Department of Clinical Pharmacology, Pharmacy and Environmental Medicine, Institute of Public Health, University of Southern Denmark, Odense, Denmark; 2 Department of Clinical Research, Faculty of Health Sciences, University of Southern Denmark, Odense, Denmark; 3 Department of Child and Adolescent Psychiatry, Mental Health Services in the Region of Southern Denmark, Odense, Denmark; 4 Department of Growth and Reproduction, Rigshospitalet, Copenhagen University Hospital, Copenhagen, Denmark; 5 International Center for Research and Research Training in Endocrine Disruption of Male Reproduction and Child Health (EDMaRC), Rigshospitalet, University of Copenhagen, Copenhagen, Denmark; 6 Hans Christian Andersen Children’s Hospital, Odense University Hospital, Odense, Denmark; 7 OPEN Patient data Explorative Network (OPEN), Odense, Denmark

**Keywords:** Bisphenol A, ADHD, Autism Spectrum Disorder, Neurodevelopment, Endocrine disruptor

## Abstract

**Background:**

Bisphenol A (BPA) is a non-persistent chemical with endocrine disrupting abilities used in a variety of consumer products. Fetal exposure to BPA is of concern due to the elevated sensitivity, which particularly relates to the developing brain. Several epidemiological studies have investigated the association between prenatal BPA exposure and neurodevelopment, but the results have been inconclusive.

**Objective:**

To assess the association between in utero exposure to BPA and Attention Deficit/Hyperactivity Disorder (ADHD-) symptoms and symptoms of Autism Spectrum Disorder (ASD) in 2 and 5-year old Danish children.

**Method:**

In the prospective Odense Child Cohort, BPA was measured in urine samples collected in gestational week 28 and adjusted for osmolality. ADHD and ASD symptoms were assessed with the use of the ADHD scale and ASD scale, respectively, derived from the Child Behaviour Checklist preschool version (CBCL/1½-5) at ages 2 and 5 years. Negative binomial and multiple logistic regression analyses were performed to investigate the association between maternal BPA exposure (continuous ln-transformed or divided into tertiles) and the relative differences in ADHD and ASD problem scores and the odds (OR) of an ADHD and autism score above the 75th percentile adjusting for maternal educational level, maternal age, pre-pregnancy BMI, parity and child age at evaluation in 658 mother-child pairs at 2 years of age for ASD-score, and 427 mother-child pairs at 5 years of age for ADHD and ASD-score.

**Results:**

BPA was detected in 85.3% of maternal urine samples even though the exposure level was low (median 1.2 ng/mL). No associations between maternal BPA exposure and ASD at age 2 years or ADHD at age 5 years were found. Trends of elevated Odds Ratios (ORs) were seen among 5 year old children within the 3rd tertile of BPA exposure with an ASD-score above the 75th percentile (OR = 1.80, 95% CI 0.97,3.32), being stronger for girls (OR = 3.17, 95% CI 1.85,9.28). A dose-response relationship was observed between BPA exposure and ASD-score at 5 years of age (p-trend 0.06) in both boys and girls, but only significant in girls (p-trend 0.03).

**Conclusion:**

Our findings suggest that prenatal BPA exposure even in low concentrations may increase the risk of ASD symptoms which may predict later social abilities. It is therefore important to follow-up these children at older ages, measure their own BPA exposure, and determine if the observed associations persist.

**Supplementary Information:**

The online version contains supplementary material available at 10.1186/s12940-021-00709-y.

## Introduction

Attention Deficit Hyperactivity Disorder (ADHD) and Autism Spectrum Disorders (ASD) are two of the most common neurodevelopmental disorders in children [1]. Both have a large impact on the lives of the affected individuals and are associated with reduced everyday life functioning, academic performance and a decreased quality of life [2, 3]. The global prevalence of ADHD is estimated to be between 5.3–7.2% [4, 5] and the European prevalence of ASD reported range from 4.2/1000–17.4/1000 with high variability in prevalence estimates worldwide [6]. Although genetic factors play an important role in the occurrence of these disorders, knowledge of the underlying causes are scarce, and environmental toxicants are suspected to contribute to their occurrence [7–10].

During fetal life, the brain development is particular sensitive, and prenatal exposure to environmental chemicals is therefore of concern [11]. Bisphenol A (BPA) is a non-persistent chemical used in the production of polycarbonate plastics and epoxy resins. It is a known endocrine disrupter, which possesses estrogenic, anti-estrogenic, anti-androgenic and anti-thyroid properties [12]. It is present in a variety of consumer products, including; microwave ovenware, food and liquid storage containers, children’s toys, protective inner lining in food cans, dental sealants, and thermal paper receipts [13]. Exposure to BPA occurs through oral ingestion of BPA contaminated food, through dermal contact or inhalation [14]. BPA is present in urine samples of 95% of the general population in the US [15], and > 80% of samples of the general population in Denmark [16], thus BPA exposure is ubiquitous. Several human studies have investigated associations between maternal BPA exposure and behavioural alterations in the offspring e.g. externalizing and internalizing behaviour, hyperactivity, inattention, anxiety, aggression and autistic symptoms [17–31]. However, the results have been inconclusive, and most cohort studies have reported generally larger effect size in boys [20–24, 27]. Furthermore, a recent study has provided evidence that prenatal BPA exposure has an adverse effect on the development of white matter microstructure in some brain regions which might mediate behavioural problems in children [29]. Three studies found no association between prenatal BPA exposure and behaviour [30–32]. The inconsistencies may be explained by methodological differences between studies, the time window of exposure assessment, the specific behavioural domains tested, the matrix of exposure assessment (urine sample or cord blood), child age (2–10 years), exposure level, and sociodemographic characteristics of the study participants [33–35].

A previous study in the Odense Child Cohort (OCC) found an association between prenatal BPA exposure and delayed language development, but no association with ADHD related symptoms at 2 years of age among 658 children [32]. The lack of an association might be attributed to the low prevalence of ADHD symptoms at 2 years, and thus a follow up at 5 years might detect symptoms that were not recognized at 2 years. We therefore aimed to investigate, if maternal exposure to BPA was associated with increased symptoms of ASD at 2 and 5 years, and with increased ADHD symptoms at 5 years in 427 mother-child pairs from the OCC.

## Materials and methods

### Study population

From 2010 to 2012, women residing in the municipality of Odense with a newly diagnosed pregnancy before 16 weeks of gestation were invited to participate in the prospective OCC, and 2874 agreed to participate [36]. Twins and mothers of non-western origin (*N* = 332) were excluded from this study.

### BPA exposure assessment

BPA was measured in maternal urine samples. An aliquot of 10–12 mL fasting urine was collected in the morning at approximately week 28 of pregnancy (range: week 26–34). Samples were stored in freezers at − 80 °C until chemical analysis [36, 37]. The first 196 samples were selected randomly from women enrolled in OCC between September 2010 and June 2011. The remaining samples were selected based on availability of questionnaire and clinical data. Urine samples were deconjugated by enzymatic hydrolysis and then analysed by an isotope dilution Turboflow-LC-MS/MS method for simultaneous determination of BPA and other phenols [16, 38]. Samples were analysed in 17 batches (5 batches in 2011 and 11 in the end of 2012) all including standards for calibration curves, about 35 cohort samples, two blanks and two control pools spiked with high and low level bisphenol A standards. The inter-day variation was ≤14%. The limit of detection (LOD) for urinary BPA was 0.12 ng/mL. There was no difference in the spiked control material between the two measuring periods [16].

To account for urinary dilution, all BPA concentrations were adjusted for osmolality. In contrast to urinary creatinine adjustment, urine osmolality is directly related to the number of particles in solution and is unaffected by the molecular weight and size of these particles [39]. Osmolality was measured by freezing point depression method with automatic cryoscopic osmometer (Osmomat®030 from Gonotec, Berlin, Germany). BPA concentrations >LOD were adjusted for urinary dilution by multiplying the individual BPA urine concentration (ng/mL) with the median osmolality for all urine samples (0.62 osm/kg) and dividing it with the osmolality (osm/kg) of the individual urine sample. The BPA concentrations below the LOD were not osmolality adjusted but were substituted by LOD/√2.

### Child behaviour checklist for ages 1½-5

The Child Behaviour Checklist; 1½-5 (CBCL/1½-5) is a parent rated questionnaire measuring emotional and behavioural problems in children between 1½ and 5 years of age. CBCL/1½-5 consists of 100 problem questions, which the parents are asked to rate on a 3-point Likert scale based on the preceding 2 months as: 0 = *not true*, 1 = *somewhat/sometimes true* or 2 = *very true/ often true*. A standardized version of CBCL/1½-5 translated into Danish [40] was sent to the parents in the OCC, when children were between 1.9–4.0 (median 2.7) and 4.9–7.0 (median 5.2) years of age.

We measured ADHD related symptoms at the DSM-oriented ADHD problem scale, extracted from CBCL/1½-5. It contains 6 questions (*cannot concentrate, hyperactive, cannot stand waiting, demands met immediately, gets into everything, quickly shifts)* with a scale-score between 0 and 12 points. Autistic symptoms were measured at the DSM-oriented ASD problem scale (also called Pervasive Developmental Problem (PDP) scale), extracted from CBCL/1½-5. It contains 13 questions *(afraid to try new things, avoids eye contact, can’t stand having things out of place, disturbed by any change in routine, does not answer, does not get along with other children, repeatedly rocks head or body, seems unresponsive to affection, shows little affection toward people, speech problem, strange behaviour, upset by new people or situations, withdrawn)* with a possible scale-score between 0 and 26 points. Results were kept as raw scores and dichotomized at the 75th percentile to identify a subclinical population, and at the 90th percentile, since a score on the DSM-ADHD scale derived from CBCL/1½-5 above the 90th percentile is a predictor of a later ADHD diagnosis [41].

### Covariates

Data on maternal education and Body Mass Index (BMI) and smoking habits were obtained through questionnaires filled in during pregnancy. Birth information, including; maternal age, parity, birth weight, gestational age, and child sex was extracted from obstetric journals. Data on child’s health including duration of breastfeeding were obtained through questionnaires filled in, when the children were 3 and 18 months of age. Maternal ethnicity was obtained through data from the municipality. Finally, information regarding parental psychiatric diagnosis was extracted from the Danish National Patient Registry.

### Statistical analysis

ADHD- and ASD scores were not normally distributed and scores of respectively below and above the 75th and 90th percentile was calculated for both scales. The 75th and 90th percentile dichotomization of the ADHD score corresponded to a score of ≥4 and ≥ 5, respectively, at 5 years of age. The 75th and 90th percentile dichotomization of the ASD score corresponded to a score of ≥3 and ≥ 4, respectively, at 2 years of age, and a score of ≥4 and ≥ 5, respectively, at 5 years. At 2 years of age, 26% of children had an ASD score ≥ 3, and were dichotomized into </≥ 75th percentile*.* At 5 years of age respectively 20 and 26% of children had a score of ≥4 on the ASD and ADHD scale. Therefore, the children were dichotomized into ≤/> the 75th for ASD score and </≥ 75th percentile for ADHD score*.* BPA concentrations were not normally distributed, and 15% of concentrations were below LOD. Thus, we both divided BPA concentrations into tertiles, and kept it as an ln-transformed continuous variable. However, it should be noted that residuals did not fit the logistic model with ln-transformed continuous BPA, and thus results should be interpreted with caution. The characteristics of the included participants were compared to the excluded participants by the use of Chi^2^ test. Differences in urinary BPA concentration, ADHD and ASD scores (below and above 75th percentile) were examined according to maternal and child characteristics using non-parametric Kruskal Wallis test (BPA) and Chi^2^ test (ADHD and ASD).

First, we used multiple logistic regression to calculate the Odds Ratio (OR) and 95% confidence intervals (95% CI) of having an ADHD or ASD problem score > 75th and ≥ 90th percentile across increasing maternal BPA exposure (continuous ln-transformed or divided into tertiles) and to test the trend across tertiles. Second, ASD- and ADHD-scores were analysed as ordinal data using negative binomial regression to estimate the relative change in ASD or ADHD-score expressed as ratio of risk between exposure groups. In addition, a sub-analysis was undertaken to calculate the odds ratio of having an ASD-score above the 75% percentile at both 2 and 5 years of age. Confounders were selected based on careful review of the literature, and if they varied by exposure and outcome status. Furthermore, covariates were included, if they changed the effect estimate of the independent variable with ≥10%. Possible confounders included in the final analyses were maternal educational level, parity, pre-pregnancy BMI and maternal age. Furthermore, we adjusted for child age at evaluation, as small differences in age could affect neuropsychological status. In addition, the unstratified analyses were also adjusted for child sex. A sensitivity analyses was performed for the 75th percentile additionally adjusting for the possible mediators breastfeeding and birth weight, as we were interested in the direct rather than the total effect of BPA on ADHD and ASD symptoms. Since previous research has found BPA to cause sexually dimorphic alterations in child behaviour [33, 34], all regression analyses were also performed for boys and girls separately, and we tested, if the associations were modified by sex by inserting interaction terms (BPA x child sex) in the regression models. No significant interaction was found between maternal BPA exposure and child sex.

To evaluate the fit of the logistic regression models, goodness of fit was tested using Hosmer-Lemeshow test and accepted for all models. Results are presented with an Odds Ratio (OR) 95% confidence interval (CI), and a *p*-value < 0.05 was considered statistically significant.

## Results

**Fig. 1:**
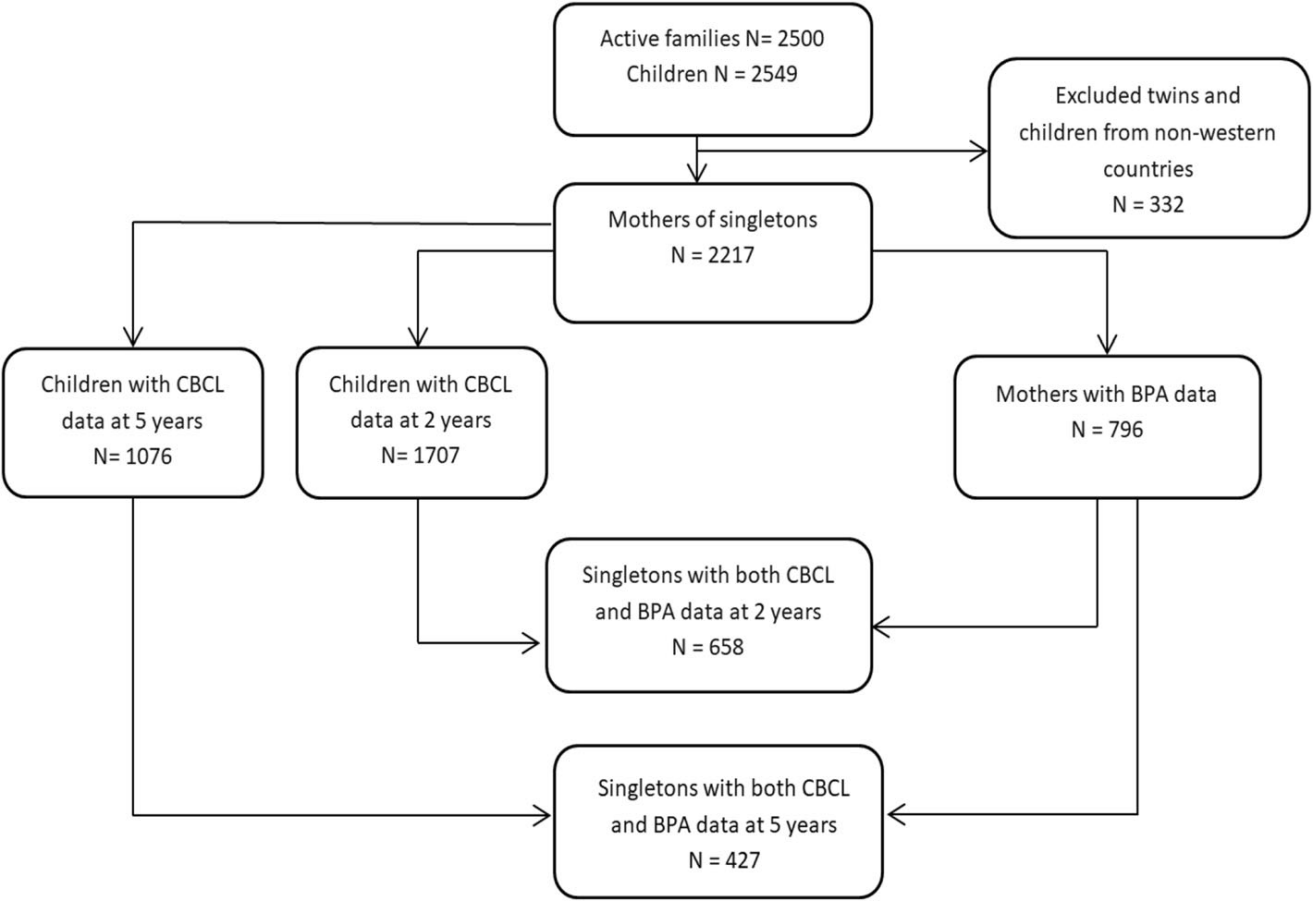
Flowchart presenting the selection of the 658 and 427 study participants from the Odense Child Cohort

A total of 2217 mother-child pairs were enrolled in the OCC at 27 months of age. Of these, 1707 responded to CBCL1½-5 at 2 years of age, 1076 participants responded at 5 years of age, and 796 mothers had BPA measured in urine. A total of 658 participants had both BPA and CBCL1½-5 data available at 2 years of age, and 427 at 5 years of age, and were therefore included in this study (Fig. 1). Compared to the excluded participants (*N* = 1559), the included participants were less often smokers and breastfeed for a longer period of time (Supplementary Table 1).

BPA was detected in 85.3% of samples with a median (25–75 percentile) concentration of 1.2 ng/mL (0.5–2.6) (Table 1). BPA concentrations were significantly higher among overweight women. Furthermore, BPA concentrations were higher among nulliparous women, among less educated women and among children born at term. Women who did not breastfeed their children exclusively also had higher BPA concentration than women who breastfed, though these differences were not statistically significant (Table 1).

**Table 1:** Maternal and child characteristics according to median (M) and 25–75 percentiles (25–75%) BPA concentrations in maternal urine; and percentage of children with an ADHD- or ASD- score above the 75th percentile at 2 or 5 years of age

***Maternal and child characteristics***	N (%)	BPA ng/mL M (25–75%)	ASD-score > 75% 2 years Percent	ASD-score > 75% 5 years Percent	ADHD-score > 75% 5 years Percent
	658 (100)	1.2 (0.5–2.6)			
**Parity**					
Nulliparous	371 (56)	1.37 (0.47–2.88)	32	42	29
Multiparous	287 (44)	1.20 (0.49–2.26)	18	24	22
*P-value*^*a*^		*0.07*	< 0.001*	< 0.001*	*0.08*
**Pre-pregnancy BMI (kg/m**^**2**^**)**					
> 18.5- < 25	412 (62)	1.03 (0.30–2.32)	27	32	24
25–30	169 (26)	1.82 (0.90–2.77)	24	33	28
> 30	77 (12)	1.17 (0.55–2.97)	24	49	33
*P-value*^*a*^		0.002*	*0.65*	*0.06*	*0.35*
**Smoking**					
No	639 (97)	1.23 (0.47–2.54)	25	32	25
Yes	19 (3)	1.21 (0.63–2.68)	37	73	33
*P-value*^*a*^		*0.19*	*0.29*	0.001*	*0.49*
**Education** ^**b**^					
high school or less	177 (27)	1.53 (0.55–2.66)	32	54	39
high school + 1–4 years	336 (53)	1.20 (0.49–2.73)	25	29	22
high school + > 4 years	131 (20)	1.35 (0.33–2.24)	18	21	18
*P-value*^*a*^		*0.34*	0.04*	< 0.001*	< 0.001*
**Age**					
< 25	61 (9)	1.08 (0.39–3.34)	40	35	44
25–34	438 (67)	1.23 (0.47–2.49)	26	35	25
> 34	159 (24)	1.28 (0.55–2.52)	19	30	21
*P-value*^*a*^		*0.82*	0.006*	*0.06*	0.03*
**Psychiatric predisposition**					
None	565 (86)	1.27 (0.50–2.52)	24	33	24
Predisposition from parents	93 (14)	1.14 (0.38–2.97)	36	42	33
*P-value*^*a*^		*0.88*	0.01*	*0.16*	*0.14*
**Birth weight (gram)**					
≤ 3560	332 (51)	1.24 (0.44–2.89)	27	42	27
> 3560	326 (49)	1.23 (0.52–2.38)	25	26	24
*P-value*^*a*^		*0.13*	*0.52*	< 0.001*	*0.5*
**Exclusive breastfeeding (weeks)**^**c**^					
0	101 (16)	1.96 (0.79–3.72)	23	45	36
1–12	256 (42)	1.20 (0.47–2.38)	25	34	31
> 12	259 (42)	1.24 (0.41–2.42)	27	32	18
*P-value*^*a*^		*0.22*	*0.65*	*0.18*	0.005*
**Gestation**					
< 37 + 0	24 (4)	0.54 (0.00–2.73)	42	62	31
> 37 + 0	634 (96)	1.23 (0.49–2.54)	25	33	26
*P-value*^*a*^		*0.84*	*0.07*	0.03*	*0.67*

^a^*P*-value < 0.05 with Kruskal wallis test (BPA) or Chi^2^ test (Autistic- or ADHD symptoms)

^b^ 4 observations missing

^c^42 observations missing

The median score (25–75%) on the ASD-scale was 1 (0–2) for girls, and 1 (0–3) for boys at 2 years of age and 1 (0–3) for girls, and 2 (1–3) for boys at 5 years of age. The median score (25–75%) on the ADHD scale was 2 (0–3) for girls, and 2 (1–4) for boys at 5 years of age. Higher ADHD/ASD scores were found among children of mothers, who were nulliparous, smokers, less educated, younger, among children with a psychiatric predisposition, children who had been exclusively breastfed for a shorter duration, children with lower birth weight, and children who were born prematurely (Table 1).

At 2 years of age, no monotonic-trend was observed across tertiles of BPA for the categorized and continuous ASD-score (Table 2, Table 3). In addition, no association was found with continuous ln-transformed BPA (Table 3). At 5 years of age, children within the 3rd tertile of BPA exposure had a 23% increase in ASD-score (IRR: 1.23 (95% CI 0.98,1.53)), compared to those in the 1st tertile (p-trend = 0.07), although not significant (Table 2). Furthermore, children within the 3rd tertile of prenatal BPA exposure had an OR of 1.80 (95% CI 0.97,3.32) scoring above the ASD-score > 75th percentile compared to those within the 1st tertile of exposure (p-trend = 0.06), and each doubling in BPA exposure (ng/mL) was associated with a 25% higher odds of an ASD-score score > 75th percentile (OR: 1.25 (95% CI 1.03,1.52)). The sex-stratified analysis showed that the association was stronger in girls, and girls in the 3rd tertile of BPA exposure had an OR of 3.17 (95% CI 1.85,9.28) and boys had an OR of 1.42 (95% CI 0.64,3.13) compared to those within the 1st tertile of exposure, and a similar direction of association was found with continuous ln-transformed BPA (Table 3). The association was even stronger for the 90th percentile cut-off in girls, (OR: 5.03 (95% CI 0.97–26.08)) (p-trend 0.04) but the confidence interval was also wider (Table 3).

**Table 2:** Negative binomial regression analysis of the association between osmolality adjusted maternal BPA exposure divided into tertiles and the incidence rate ratio (IRR) and 95% confidence intervals (CI 95%) of ADHD-score in boys and girls at 2 and 5 years of age

**Osmolality adjusted BPA (ng/mL)**	**Adjusted**^**a**^ **IRR (CI 95%)** **All (*N*** **= 654)**	**Adjusted**^**a**^ **IRR (CI 95%) Boys (*N*** **= 347)**	**Adjusted**^**a**^ **IRR (CI 95%) Girls (*N*** **= 307)**
**ASD-score at 2 years**			
**1th tertile (≤0.87)**	Reference	Reference	Reference
**2nd tertile (0.88–1.96)**	0.87 (0.71–1.07)	0.85 (0.65–1.10)	0.89 (0.64–1.24)
**3rd tertile (≥ 1.97)**	1.02 (0.84–1.24)	1.08 (0.84–1.39)	0.91 (0.66–1.25)
**p-trend**^**b**^	0.80	0.48	0.54
**Osmolality adjusted BPA (ng/mL)**	**Adjusted**^**a**^ **IRR (CI 95%)** **All (*N*** **= 425)**	**Adjusted**^**a**^ **IRR (CI 95%) Boys (*N*** **= 223)**	**Adjusted**^**a**^ **IRR (CI 95%) Girls (*N*** **= 202)**
**ASD-score 5 years**			
**1st tertile (≤0.87)**	Reference	Reference	Reference
**2nd tertile (0.88–1.96)**	1.13 (0.89–1.42)	1.09 (0.80–1.51)	1.18 (0.58–5.54)
**3rd tertile (≥ 1.97)**	1.23 (0.98–1.53)	1.20 (0.88–1.63)	1.29 (0.93–1.78)
**p-trend**^**b**^	0.07	0.24	0.13
**Osmolality adjusted BPA (ng/mL)**	**Adjusted**^**a**^ **IRR (CI 95%)** **All (*N*** **= 425)**	**Adjusted**^**a**^ **IRR (CI 95%) Boys (*N*** **= 223)**	**Adjusted**^**a**^ **IRR (CI 95%) Girls (*N*** **= 202)**
**ADHD-score at 5 years**			
**1st tertile (≤0.87)**	Reference	Reference	Reference
**2nd tertile (0.88–1.96)**	0.96 (0.76–1.22)	0.88 (0.66–1-17)	1.11 (0.75–1.65)
**3rd tertile (≥ 1.97)**	1.08 (0.86–1.35)	0.98 (0.75–1.30)	1.26 (0.87–1.85)
**p-trend**^**b**^	0.86	0.43	0.76

^a^Analyses adjusted for maternal education, maternal age, pre-pregnancy BMI, child age at evaluation, parity. The sex-combined analyses were additionally adjusted for child sex

^b^Trend across tertiles tested by inserting the tertile osmolality adjusted BPA as an ordinal indicator variable (0,1,2)

* *P*-value < 0.05

**Table 3:** Multiple logistic regression analysis of the association between osmolality adjusted maternal BPA exposure divided into tertiles and as continuous ln-transformed and the odds ratio (OR) and 95% confidence intervals (CI 95%) of an ASD-score > 75th percentile compared to < 75th percentile (reference) and ≥ 90th percentile compared to <90th percentile (reference) in boys and girls at 2 and or 5 years of age

	**≥75th percentile** **Adjusted OR (CI 95%)**			**≥90th percentile** **Adjusted OR (CI 95%)**		
**ASD-score 2 years**						
**Osmolality adjusted BPA (ng/mL)**	**All** **(*N*** **= 166/488)**	**Boys** **(*N*** **= 97/250)**	**Girls** **(*N*** **= 69/238)**	**All** **(*N*** **= 98/556)**	**Boys** **(*N*** **= 64/283)**	**Girls** **(*N*** **= 34/273)**
**1th tertile (≤0.87)**	Reference	Reference	Reference	Reference	Reference	Reference
**2nd tertile** **(0.88–1.96)**	0.81 (0.47–1.20)	0.86 (0.47–1.59)	0.71 (0.35–1.43)	0.84 (0.47–1.48)	0.80 (0.39–1.66)	0.89 (0.35–2.27)
**3rd tertile** **(≥ 1.97)**	1.06 (0.70–1.68)	1.07 (0.60–1.94)	1.05 (0.54–2.31)	1.18 (0.69–2.00)	1.37 (0.70–2.68)	0.83 (0.34–2.06)
**p-trend**^**b**^	0.65	0.78	0.86	0.49	0.31	0.69
**Continuous** **lnBPA**^**c**^	1.08 (0.94–1.24)	1.11 (0.93–1.34)	1.02 (0.83–1.25)	1.10 (0.93–1.31)	1.17 (0.94–1.45)	0.97 (0.73–1.29)
	**>75th percentile** **Adjusted OR (CI 95%)**			**≥90th percentile** **Adjusted OR (CI 95%)**		
**ASD-score 5 years**						
**Osmolality adjusted BPA (ng/mL)**	**All** **(*N*** **= 86/339)**	**Boys** **(*N*** **= 54/169)**	**Girls** **(*N*** **= 32/170)**	**All** **(*N*** **= 59/366)**	**Boys** **(*N*** **= 34/189)**	**Girls** **(*N*** **= 25/177)**
**1th tertile (≤0.87)**	Reference	Reference	Reference	Reference	Reference	Reference
**2nd tertile** **(0.88–1.96)**	1.22 (0.63–2.36)	1.02 (0.43–2.40)	1.80 (0.58–5.54)	0.86 (0.36–1.99)	0.68 (0.24–1.90)	1.96 (0.32–11.83)
**3rd tertile** **(≥ 1.97)**	1.80 (0.97–3.32)	1.42 (0.64–3.13)	3.17 (1.85–9.28)*	1.65 (0.78–3.49)	1.20 (0.47–2.99)	5.03 (0.97–26.08)
**p-trend**^**b**^	0.06	0.37	0.03*	0.15	0.69	0.04*
**Continuous** **lnBPA**^**c**^	1.25 (1.03–1.52)*	1.16 (0.91–1.49)	1.48 (1.04–2.12)*	1.20 (0.94–1.53)	1.10 (0.83–1.47)	1.61 (0.96–2.69)

^a^Analyses adjusted for maternal education, maternal age, pre-pregnancy BMI, child age at evaluation, parity. The sex-combined analyses were additionally adjusted for child sex

^b^Trend across tertiles tested by inserting the tertile osmolality adjusted BPA as an ordinal indicator variable (0,1,2)

^c^BPA inserted as a continuous variable transformed by the natural logarithm

* *P*-value < 0.05

Forty children had an ASD-score **≥** 75th percentile at both 2 and 5 years. Increased odds of an ASD-score **≥** 75th percentile was found among children in the 3rd tertile of prenatal BPA exposure, with an OR of 2.05 (95% CI 0.90,4.64) (p-trend = 0.06). For each doubling in BPA exposure the odds of a score *≥* 75th percentile increased with 38% (OR: 1.38 (95% CI 1.02,1.84)) compared to a score < 75th percentile (Table 4).

**Table 4:** Multiple logistic regression analysis of the association between osmolality adjusted maternal BPA exposure divided into tertiles and as continuous ln-transformed and the odds ratio (OR) and 95% confidence intervals (CI 95%) of an ASD-score ≥ 75th percentile compared to < 75th percentile (reference) in boys and girls at 2 and 5 years of age

Osmolality adjusted BPA (ng/ml)	All (***N*** = 38/376) Adjusted^a^ OR (CI 95%)	Boys (***N*** = 25/197) Adjusted^a^ OR (CI 95%)	Girls (***N*** = 13/179) Adjusted^a^ OR (CI 95%)
**ASD-score ≥ 75th percentile at both 2 and 5 years**			
**1th tertile (≤0.87)**	Reference	Reference	Reference
**2nd tertile (0.88–1.96)**	0.72 (0.26–2.01)	0.58 (0.15–2.17)	0.95 (0.17–5.52)
**3rd tertile (≥ 1.97)**	2.05 (0.90–4.64)	2.17 (0.79–6.01)	1.94 (0.44–8.57)
**p-trend**^**b**^	0.06	0.09	0.33
**Continuous lnBPA** ^**c**^	1.38 (1.02–1.84)*	1.42 (0.99–2.02)	1.34 (0.79–2.25)

^a^Analyses adjusted for maternal education, maternal age, pre-pregnancy BMI, child age at evaluation, parity. The sex-combined analyses were additionally adjusted for child sex

^b^Trend across tertiles tested by inserting the tertile osmolality adjusted BPA as an ordinal indicator variable (0,1,2)

^c^BPA inserted as a continuous variable transformed by the natural logarithm

* *P*-value < 0.05

An increase in OR was found among children in the 3rd tertile of BPA exposure and ADHD symptoms at age 5 years at the 75th percentile and at the 90th percentile cut-off (Table 5), although modest in magnitude and not statistically significant.

**Table 5:** Multiple logistic regression analysis of the association between osmolality adjusted maternal BPA exposure divided into tertiles and as continuous ln-transformed and the odds ratio (OR) and 95% confidence intervals (CI 95%) of an ADHD-score ≥ 75% compared to < 75% (reference) and ≥ 90th percentile compared to <90th percentile (reference) in boys and girls at 5 years of age

Osmolality adjusted BPA (ng/mL)	All chidren (***N*** = 109/316) Adjusted^a^ OR (CI 95%)	Boys (***N*** = 66/157) Adjusted^a^ OR (CI 95%)	Girls (***N*** = 43/159) Adjusted^a^ OR (CI 95%)
**ADHD-score 5 years ≥ 75th percentile**			
**1st tertile (≤0.87)**	Reference	Reference	reference
**2nd tertile (0.88–1.96)**	0.81 (0.45–1.45)	0.80 (0.37–1-73)	0.88 (0.36–2.20)
**3rd tertile (≥ 1.97)**	1.09 (0.64–1.87)	1.06 (0.51–2.17)	1.29 (0.57–2.99)
**P-trend**^**b**^	0.71	0.86	0.52
**Continuous lnBPA**^**c**^	1.05 (0.88–1.25)	1.01 (0.80–1.26)	1.14 (0.85–1.50)
**Osmolality adjusted BPA (ng/mL)**	**All chidren (*N*** **= 64/361)** **Adjusted**^**a**^ **OR (CI 95%)**	**Boys (*N*** **= 39/184)** **Adjusted**^**a**^ **OR (CI 95%)**	**Girls (*N*** **= 25/177)** **Adjusted**^**a**^ **OR (CI 95%)**
**ADHD-score 5 years ≥ 90th percentile**			
**1st tertile (≤0.87)**	Reference	Reference	Reference
**2nd tertile (0.88–1.96)**	0.92 (0.42–1.93)	0.60 (0.21–1.69)	1.78 (0.55–5.73)
**3rd tertile (≥ 1.97)**	1.33 (0.68–2.61)	1.45 (0.61–3.45)	1.29 (0.46–4.64)
**p-trend**^**b**^	0.37	0.32	0.56
**Continuous lnBPA**^**c**^	1.17 (0.93–1.46)	1.17 (0.88–1.56)	1.25 (0.85–1.85)

^a^Analyses adjusted for maternal education, maternal age, pre-pregnancy BMI, child age at evaluation, parity. The sex-combined analyses were additionally adjusted for child sex

^b^Trend across tertiles tested by inserting the tertile osmolality adjusted BPA as an ordinal indicator variable (0,1,2)

^c^BPA inserted as a continuous variable transformed by the natural logarithm

* *P*-value < 0.05

In the sensitivity analysis additionally adjusted for breastfeeding and birth weight the ORs did not change substantially (supplementary Table 2).

## Discussion

In this low exposed mother child cohort, maternal exposure to BPA was associated with an increased odds of an ASD-score > 75th percentile among children at 5 years of age. No association between maternal BPA exposure and ASD or ADHD scores at respectively age 2 and 5 years was found. It is important to note, that none of these children were assessed or diagnosed with ASD or ADHD and CBCL/1½-5 is not a diagnostic tool. However, the scores obtained from the scales are in accordance with the Danish reference norms [40], and previous research has established that the CBCL derived DSM-ADHD and DSM-ASD scales are both valid assessors of ADHD or ASD psychopathology [41, 42]. The lack of an association at age 2 years may be explained by CBCL/1½-5 ASD scale does not capture autism specific symptoms as well at age 2 years compared to age 5 years.

The association between maternal BPA exposure and child neurodevelopment has been investigated by several studies (reviewed in [33–35]). To the best of our knowledge, five studies have investigated the association between prenatal exposure to BPA and social impairment or autism-symptoms [26, 27, 30, 31, 43]. Two North American prospective cohort studies and one North American prospective high-ASD risk cohort found no association between maternal BPA exposure and ASD or non-typical development at 3 years [43], autistic symptoms in preschool children [30] or social impairment in school-aged children [31], which is in contrast to our findings. However, in the latter study of school aged children *Miodovnik* et al. (2011) [31] a significant association between prenatal BPA exposure and total SRS-score was found when 6 outliers were removed. Our findings are in accordance with a Korean prospective cohort with 304 mother-child pairs, which found decreased social communication skills in 4-year old girls with prenatal BPA exposure > 3.0 μg/g (creatinine adjusted) [26]. However, the American and Korean studies included multiethnic or Korean participants most with higher BPA exposure, whereas our participants were homogeneous and most of Western origin. Since ethnic origin may impact how parents rate child behaviour [44, 45], our findings are not directly comparable, but are in the same direction. A Canadian cohort study comparable to ours in terms of sociodemographic characteristics of the study population and BPA exposure [27] found an association between prenatal BPA exposure and poorer reciprocal social behaviours in 537 children, strongest in boys, measured with the Social Responsiveness Scale 2 (SRS-2) at 3 years [27]. They measured BPA at 12 weeks of gestation with a median concentration of 0.8 ng/mL, whereas we measured BPA at 28 weeks of gestation, and the median BPA concentration was 1.2 ng/mL. We found stronger associations in girls, whereas *Braun* et al. (2017) [27] found strongest associations in boys. ASD is several times more frequent in boys compared to girls [46], and in animal studies, BPA exposure has been shown to affect sexual differentiation of the brain and can either increase, decrease or eliminate sex-differences [33]. During fetal life sex steroids are crucial for the sexual differentiation of the brain, and the most vulnerable time for the sexual differentiation is assumed to be between week 8–24 of gestation [33, 47]. The difference in the sex-specific direction of the association between our study and the study by *Braun* et al. (2017) [27] might be attributed to the difference in time of exposure assessment, as early pregnancy might impact behavior in a different way as opposed to mid pregnancy. Furthermore, *Lim* et al. (2017) [26] also found stronger associations in girls and measured BPA exposure between 14 and 27 (median 20 weeks) of gestation. In our study, only 33 girls scored above the 75th percentile, which reduced power. In addition, different tests were used.

In a previous publication from our cohort, no association between maternal BPA exposure and ADHD symptoms at age 2 years was found [32]. These findings were confirmed at age 5 years in the current work, and are in contrast to the results of a large exposome study including data from 5 European cohorts that found prenatal BPA to be associated with worse externalizing behavior problems measured with the Strength and Difficulties Questionnaire between 3 and 7 years of age [48]. In addition, four studies comparable to our have investigated prenatal BPA exposure and ADHD related symptoms, and the results were conflicting [20, 21, 25, 27]. A French [25] cohort study found an association between prenatal BPA exposure and ADHD symptoms in boys at age 5 years, measured by the Strength and Difficulties Questionnaire, and a Spanish [20] cohort study found increased inattention in boys and decreased inattention in girls at 4 years of age measured with the Criteria of Diagnostic and Statistical Manual of Mental Disorders-4th Edition (ADHD-DSM-IV). BPA exposure was, however, higher than in our cohort, which could explain, why we found no associations [20, 25]. A Canadian [27] and an American study [21] with BPA exposure concentrations comparable to ours found no association between prenatal BPA exposure and ADHD symptoms at age 3 years measured with the Behaviour Assessment System for Children 2 (BASC-2), and at 6–10 years of age as measured with CBCL (school age version), respectively, which is in accordance with our findings. Furthermore in a Canadian [29] birth cohort maternal BPA exposure measured in urine samples in the 2nd trimester (median 1.5 ng/mL) was associated with increased internalizing but not externalizing behaviour problems measured with the CBCL at 2–5 years of age in 56 mother child pairs [29]. The study is not directly comparable to ours, as they measured internalizing and externalizing problems, and we only measured specific ADHD- and ASD symptoms, but our results are in the same direction. In addition, *Grohs* et al (2020) [29] suggested that the association was mediated by less developed white matter microstructure in the splenium measured with diffusion magnetic resonance imaging (MRI) scan in the 56 children who provided both prenatal BPA and CBCL data.

The ability of in utero BPA exposure to alter behaviour has been confirmed in several animal studies (reviewed in [49]). In rodents, gestational or perinatal exposure to BPA have been associated with increased anxiety-like behaviour in male [50] and female mice [51], increased hyperactivity [52] and increased anxiety-like behaviour in both male and female mice [53]. The exact mechanism by which BPA impact behaviour is not completely understood, but in vitro and in vivo studies have found that exposure to BPA may modify normal brain development [54–57] Neurodevelopment is a complex process that starts early in the embryonic stage, and disruption of the critical developmental processes such as cell proliferation, neural migration, differentiation and synaptogenesis might cause adverse effects on the developing brain and could result in neurobehavioural disorders later in life [58]. A recent overview of the adverse effects of BPA exposure suggest that by disruption of thyroid and estrogenic pathways BPA may alter Brain-Derived Neurotropic Factor (BDNF) levels, which could be an important link between BPA exposure and altered neurodevelopment [59]. BDNF is a member of the neurotrophin family of proteins and is involved in modulating neurite outgrowth, synapse plasticity, and the promotion of neuronal survival and protection [59]. In utero BPA exposure has been linked to DNA methylation changes in the transcriptionally relevant region of the BDNF gene in mice [60], and in vitro, alone and in co-exposure with other chemicals BPA increases BDNF-levels which may be linked to cellular changes (increased number of neurons and altered synaptogenises) seen in children with ASD [61]. Furthermore, elevated blood BDNF levels are seen in children with ASD and thus BDNF is a possible biomarker for ASD [62].

Our study presents several strengths, including the relatively large study population and the prospective design. Furthermore, we were able to adjust for potential confounding factors. However, some limitations need mentioning. First, only a single urine sample was used as a proxy for fetal exposure to BPA. BPA is rapidly conjugated in the human body and almost fully excreted in urine within 24 h, and therefore temporal and diurnal variation in exposure patterns and excretion rates may occur [63–65]. BPA has a high within-subject variability [65, 66]. Thus, timing of urine collection may influence the observed BPA concentration, and a single spot urine sample may not reflect average gestational exposure [66]. Obtaining more than one urine sample could have improved precision. Furthermore, we measured BPA in fasting morning samples, which may have contributed to lower BPA concentrations. Misclassification of BPA exposure is, however, not likely to be differentially associated with CBCL results, as the women were unaware of their BPA exposure, when they responded to the CBCL questionnaire, and the strengths of the associations might therefore be underestimated, which could explain the non-significant findings. Likewise, the use of a parent rated questionnaire (CBCL/1½-5) to assess ASD and ADHD symptoms may cause parental reporting bias, as parents may under- or overestimate the severity of symptoms. Misclassification of ADHD- and autistic symptoms is, however, also likely non-differential, as women were unaware of their BPA exposure when responding to the questionnaires, thereby likely making the association go towards the null-hypothesis and explain the lack of statistically significant findings in our study.

Even though we adjusted for potential confounders, the possibility of additional confounding from e.g. maternal IQ cannot be dismissed. Furthermore, it is possible that other environmental chemicals in co-exposure with BPA could impact the findings. Finally, BPA was measured in gestational week 28, and brain development may be more vulnerable during early or late pregnancy or even during childhood, as studies have found childhood (preschool) BPA exposure is associated with behavioural alterations in children [18, 19, 22–24]. Thus, the association between BPA exposure and child behaviour could be dependent on the developmental time-window of exposure assessment. Unfortunately, we did not measure postnatal BPA exposure in children from the OCC. Future studies are needed to identify periods of heightened vulnerability to BPA exposure during fetal and childhood development.

## Conclusions

In conclusion, in this low exposed mother-child cohort, children of mothers within the highest tertile of BPA exposure had suggestive higher odds of having an ASD-score > 75th percentile at 5-years of age whereas no association was found at age 2 years. High scores may predict later ASD symptoms which may influence social and learning abilities, and it is therefore important to follow-up these children and measure their own BPA exposure to determine if these findings persist.

## Supplementary Information

**Additional file 1: Supplementary table 1.** Maternal and child characteristics according to included participants with BPA and CBCL1½-5 data at 2 years (*N*=658) and excluded participants (*N*=1559). **Supplementary table 2.** Multiple logistic regression analysis of the association between osmolality adjusted maternal BPA exposure divided into tertiles and the odds ratio (OR) and 95% confidence intervals (CI 95%) of an ASD and ADHD-score >75% compared to <75% (reference) at 2 and/or 5 years of age.

## Data Availability

The datasets used and analysed during the current study are available from the corresponding author on reasonable request.

## Abbreviations

°C: Degrees celcius
ADHD: Attention Deficit Hyperactivity Disorder
ASD: Autism Spectrum Disorder
BMI: Body Mass Index
BPA: Bisphenol A
CBCL: Child Behaviour Checklist
CI: Confidence interval
IRR: Incidence rate ratio
LC-MS/MS: Liquid chromatography and triple quadrupole mass spectrometry
LOD: Limit of Detection
mL: mililitre
ng: nano gram
OCC: Odense Child Cohort
OR: Odds ratio
