# Supplementary material for: Prenatal exposure to bisphenol A and autistic- and ADHD-related symptoms in children aged 2 and5 years from the Odense Child Cohort
Source: Environ Health. 2021 Mar 12;20:24. doi: 10.1186/s12940-021-00709-y (PMC7955642; doi:10.1186/s12940-021-00709-y)
Supplement: Supplementary file 1 — Additional file 1: Supplementary table 1. Maternal and child characteristics according to included participants with BPA and CBCL1½-5 data at 2 years (N=658) and excluded participants (N=1559). Supplementary table 2. Multiple logistic regression analysis of the association between osmolality adjusted maternal BPA exposure divided into tertiles and the odds ratio (OR) and 95% confidence intervals (CI 95%) of an ASD and ADHD-score >75% compared to <75% (reference) at 2 and/or 5 years of age. [file 12940_2021_709_MOESM1_ESM.docx]

*Supplementary table 1: Maternal and child characteristics according to included participants with BPA and CBCL1½-5 data at 2 years (N=658) and excluded participants (N=1559)*

|  | ***Included participants(n=658)/Excluded participants (n=1559)***  ***%*** |
| --- | --- |
| **Smoking**  No  Yes  *p-value* | 30/70  17/83  ***0.002*** |
| **Education^a^**  high school or less  high school +1-4 years  high school + >4 years  *p-value* | 28/72  31/69  30/70  *0.25* |
| **Parity**  Nulliparous  Multiparous  *p-value* | 30/70  29/71  *0.57* |
| **Pre-pregnancy BMI (kg/m^2^)^d^**  >18.5-24.99  25-30  >30  *p-value* | 28/72  33/67  30/70  *0.11* |
| **Age^e^**  <25  25-34  >34  *p-value* | 29/71  30/70  30/70  *0.97* |
| **Birth weight (grams)^f^**  ≤ 3545  >3545  *p-value* | 30/70  29/71  *0.82* |
| **Psychiatric predisposition**  None  Predisposition from parents  *p-value* | 31/69  25/75  *0.05* |
| **Sex**  Boy  Girl  *p-value* | 30/70  30/70  *0.98* |
| **Gestation^h^**  < 37+0  > 37+0  *p-value* | 28/72  30/70  *0.70* |
| **Breastfeeding (weeks)^b^**  0  1-12  >12  *p-value* | 30/70  35/65  28/72  ***0.01*** |

*P-value <0.05 with Chi^2^ test, ^a^= 41 missing observations, ^b^= 215 missing observations*

*Supplementary table 2. Multiple logistic regression analysis of the association between osmolality adjusted maternal BPA exposure divided into tertiles and the odds ratio (OR) and 95% confidence intervals (CI 95%) of an ASD and ADHD-score >75% compared to <75% (reference) at 2 and/or 5 years of age.*

| **ASD or ADHD score >75^th^ percentile** | | |
| --- | --- | --- |
| **Osmolality adjusted BPA (ng/mL) Adjusted^a^ OR (CI 95%)**  **ASD-2 years (N=612)** | **Adjusted^a^ OR (CI 95%) ASD 5 years (N=402)** | **Adjusted^a^ OR (CI 95%) ADHD 5 years (N = 402)** |
| 1st tertile (≤0.87) Reference | Reference | Reference |
| 2nd tertile (0.88-1.96) 0.82 (0.53-1.26) | 1.12 (0.56-2.59) | 0.79 (0.43-1.44) |
| 3rd tertile (≥ 1.97) 1.03 (0.67-1.60) | 1.89 (0.99-3.58) | 1.07 (0.61-1.87) |
| p-trend^b^  0.86 | 0.43 | 0.76 |

*^a^Analyses adjusted for maternal education, maternal age, pre-pregnancy BMI, child age at evaluation, parity, child sex , breastfeeding and birth weigth*

^b^*Trend across tertiles tested by inserting the tertile osmolality adjusted BPA as an ordinal variable (0,1,2).*

** P-value < 0.05*
